# 

**Published:** 1921-02-19

**Authors:** 


Royal Victoria Infirmary, Newcastle-on-Tyne,

### Forthcoming Events.

The Mental After-Care Association.—Annual Meeting on Wednesday, February 23, at 3 p.m., at Bridewell Royal Hospital, New Bridge Street, E.C. The chair will be taken by Sir Charles Cheers Wakefield, Bart., Alderman, C.B.E. (President of Bridewell and Bethlem Royal Hospitals). The following have kindly consented to speak: The Right Rev. the Lord Bishop of Barking, D.D., the Right Hon. Sir William Byrne, Bart., K.C.V.O., C.B. (Chairman of the Board of Control), the Hon. John Mansfield (Lord Chancellor's Visitor in Lunacy), Dr. Nathan Raw, C.M.G., M.P.

### Matrons' and Sisters' Appointments.

Chalfont and Gerrards Cross Cottage Hospital.—Miss F. M. Crooks has been appointed matron. She was trained at Walsall General Hospital, and for three years has nursed under the Overseas Nursing Association. She has the certificate of the Central Midwives Board and of the Chartered Society of Massage and Medical Gymnastics.

Manchester Babies' Hospital.—Miss Margaret Jones has been appointed matron. She was trained at Ashton-under-Lyne District Infirmary and Children's Hospital, and then became successively sister at Bradford Children's Hospital, sister and assistant matron of Ancoats Hospital, Manchester, and matron of Ancoats Hospital Convalescent Home. During 1914 to 1918 Miss Jones was doing military nursing.

Cameron Hospital, West Hartlepool.—Miss Sara Shaw has been appointed sister. She was trained at the County Hospital, York.

Royal Cornwall Sailors' Hospital, Falmouth.—Miss Helen Mair has been appointed sister. She was trained at the Royal Infirmary, Edinburgh, and under the Territorial Force Nursing Service, was sister-in-charge of surgical wards and casualty clearing-stations in France, and later with the British Army on the Rhine.

Glenafton Sanatorium, New Cumnock.—Miss E. H. Gordon has been appointed matron. She was trained at the Royal Victoria Infirmary, Newcastle-on-Tyne, and is at present matron of the Craiglea Hospital for Officers, Troon. Miss Gordon's previous appointments have been sister at Park Fever Hospital, Hither Green; matron of Moreton Paddox Military Hospital and matron of Cornekia, Lady Wimborne's Hospital, Roehampton. She has also engaged in private nursing.

Bromborough Pool Maternity Hospital, Port Sunlight.—Miss F. M. Garrett has been appointed matron. She was trained at Croydon Borough Hospital and Greenwich Infirmary, taking district training also in London. She has twice held the appointment of sister at the North-Eastern Hospital, Tottenham, has been assistant matron and temporary matron of the Maternity Hospital, Sta. Luiz, Brazil, and matron of the Mission of Home Maternity Home, Anerley.

St. Catherine's Hospital, Cawnpore.—Miss Mary Hall Simpson has been appointed sister of this hospital under the Society for the Propagation of the Gospel in Foreign Parts. Miss Simpson was trained at the Derbyshire Royal Infirmary and at the Jessop Hospital, Sheffield, where she obtained her C.M.B. She took her missionary training at St. Michael's College, Wantage. Miss Simpson sails for India on March 11.

Ty Bryn Infirmary, Tredegar.—Miss Edith Bremerton has been appointed sister. She was trained at the Warneford Hospital, Leamington, and has since been engaged in district nursing in Yorkshire and in private nursing. She has the certificate of the Central Midwives Board, and is a member of the College of Nursing.

Lincolnshire Nursing Association.—Miss Grace Wright has been appointed county superintendent. She was trained at Bristol Royal Infirmary, and has been county superintendent of Oxfordshire. Miss Wright served as sister in the Territorial Force Nursing Service from 1914 to 1919 at the 2nd Eastern General Hospital, Brighton, and the 54th General Hospital, France.

The Infirmary, Holgate, Linthorpe, Middlesbrough.—Miss H. L. Walker has been appointed sister at this hospital, where she was trained and has served as ward sister. She has also held the post of staff nurse at the Women and Children's Hospital, Leeds. She took her midwifery training at the Birmingham Maternity Hospital.

Ware County Tuberculosis Hospital.—Miss E. A. Cromer has been appointed sister. She was trained at the Leeds General Infirmary, where she was later sister. She has also been home and theatre sister at Bury Infirmary, and sister in the Territorial Force Nursing Service from 1914 to 1919. She has had nursing experience in the United States.

District Hospital, West Bromwich.—Miss F. I. Hardy has been appointed sister for the orthopædic department. She was trained at St. George's Hospital, and has been masseuse nurse at the National Hospital, East Finchley, sister Q.A.I.M.N.S.R. in France and at home, and massage sister at the General Hospital, Northampton.

### Recent Official Publications.

(To be obtained at H.M. Stationery Office, Imperial House, Kingsway, W.C. 2. The prices include postage.)

Midwives Acts 1902 and 1916. Report on Work of C.M.B. for year ended March 31, 1920. 3d.

Board of Education—No. 2336. Regulations for Secondary Schools in Wales, July 1918. 3d.

Census Regulations 1920—Provisional and Draft, December 21 and 24, 1920. Each post free 11½d.

Housing Journal No. 39, January 3, 1921. 4d.

Registrar-General of Births, Deaths, and Marriages Supplement to Seventy-fifth Annual Report, Part II. Abridged Life Tables (Cmd. 1010). Post free 1s. 9½d. Eighty-second Annual Report, 1919 (Cmd. 1017). 8s.

Poison England and Scotland, No. 2263. Order of Council, December 1, 1920. 1½d.

Medical Research Council for year 1919-20 (Cmd. 1088). 11d.

National Physical Laboratory. Collected Researches, Vol. XV., 1920. 21s.

National Health Insurance Act. Amendment Order, December 21, 1920. 1½d. Draft Regulations, January 4, 1921, proposed to be made by the Minister of Health, amending Medical Benefit Regulations, 1920. 1½d. Provisional Regulations, December 31, 1920. 1½d.

Civil Service National Whitley Council. Report of Cost of Living Committee (Cmd. 1107). 1½d.

Medical Research Council. Special Report Series No. 57. Studies in Wound Infections. 4s. 9d.

Medical Profession. Order in Council, December 21, 1920, revoking Order in Council applying to Kingdom of Belgium. 1½d.

Unemployment Insurance (Associations). Supplementary Regulations, December 31, 1920. 1½d.

Ministry of Health: Memo. 39/Food. Steps to be taken by Medical Officers of Health in Cases of Suspected Food Poisoning. 1½d.

### The Book Market.

The receipt of the following is acknowledged:—

H. Kimpton: "Practical Text-Book of Midwifery." By Robert Jardine, M.D., M.R.C.S., etc. Seventh edition. 7s. 6d. net.

Messrs. Christopher, 22 Berners Street, W. 1: "A Consulting Surgeon in the Near East." By Lieut.-Col. A. H. Tubby, C.B., C.M.G., M.S., F.R.C.S., etc., etc. 15s. net.

G. Bell & Sons, Ltd.: "The World of Sound." By Sir Wm. Bragg, K.B.E., F.R.S., D.Sc. 6s. net.

Maclehose Jackson & Co., Glasgow: "Memories and Musings of a Hospital Surgeon." 7s. 6d. net.

G. P. Putnam's Sons: "A Short History of England." By L. L. Dock, R.N., and J. M. Stewart, A.M., R.N. 17s. 6d.

W. Heinemann, Ltd.: "Gout: Its Etiology, Pathology, and Treatment." By L. J. Llewellyn, M.B. 30s. net. "Diseases of the Throat, Nose, and Ear." By D. McKenzie, M.D., F.R.C.S.E. 42s. net.

Service Publications, Ltd., 115 Fleet Street, E.C. 4: "The Service Handbook." By Capt. H. H. C. Baird, D.S.O. 2s. 6d.

H. K. Lewis & Co., Ltd.: "A Guide to Anatomy." By E. D. Ewart. 16s. net. "Infant Weight Chart for Daily Records" (1 oz. scale). 2s. 6d. net.

A. & C. Black, Ltd.: "General Practice and X-Rays." By A. V. Knox, M.B., B.Ch., and R. Knox, M.D., C.M., M.R.C.S., L.R.C.P. 15s. net.

J. Bale, Sons & Danielsson, Ltd.: "The Diseases of the New-born." By Dr. August Ritter von Reuss. 52s. 6d. net.

E. & S. Livingstone: "A Pocket Book of Ophthalmology." By A. J. Ballantyne, M.D. 5s. 6d. net.

